# A Review of the Long-Term Cardiovascular Consequences of Hypertensive Disorders of Pregnancy

**DOI:** 10.7759/cureus.100288

**Published:** 2025-12-28

**Authors:** Ahmed Rudwan, Leena Saeed, Layla Mahir, Aya Husameldin, Ammar Ahmed, Qubaa A Elzubair, Waad Ahmed, Muqadsa Malik, Enaam Rudwan

**Affiliations:** 1 Research, Hamad Medical Corporation, Doha, QAT; 2 General Medicine, Ministry of Public Health, Doha, QAT; 3 Medicine and Surgery, National Ribat University, Doha, QAT; 4 Medicine and Surgery, Istanbul Okan University, Istanbul, TUR; 5 Medicine and Surgery, Near East University, Nicosia, CYP; 6 Health, Qatar University, Doha, QAT; 7 Medicine, Ahfad University, Khartoum, SDN; 8 Medicine, Foundation University, Rawalpindi, PAK; 9 Obstetrics and Gynecology, Hamad Medical Corporation, Doha, QAT

**Keywords:** cardiovascular disease, hypertensive disorders of pregnancy, long-term risk, preeclampsia, women’s health

## Abstract

Hypertensive disorders of pregnancy (HDP) are a major worldwide issue, affecting 5-10% of pregnancies and causing feto-maternal mortality and morbidity. It includes chronic, gestational hypertension, preeclampsia, and chronic hypertension with preeclampsia. The underlying pathophysiology includes aberrant placental perfusion, angiogenic imbalance, endothelial dysfunction, and hereditary susceptibility. Women with HDP face a markedly increased risk of cardiovascular disease, chronic renal disease, and metabolic syndrome, with larger connections than those with gestational diabetes. Measures such as early aspirin prophylaxis, lifestyle modifications, and prompt antihypertensive therapy are beneficial, but adherence remains low. Postpartum monitoring is generally underused, despite its importance in reducing future cardiovascular risk. Addressing these gaps is critical for lowering maternal mortality, avoiding recurring problems, and improving health outcomes.

## Introduction and background

Hypertension is a frequent health problem during pregnancy, occurring in 5% to 10% of pregnancies, and is a contributing factor to maternal mortality in industrialized countries. Between 1998 and 2006, the incidence of hypertension during delivery hospitalizations rose from 67.2 to 81.4 per 1000 deliveries, owing in part to the increasing incidence of cardiovascular and metabolic illness in reproductive age. Mothers over the age of 40, obesity before pregnancy, excessive weight gain, and gestational diabetes are all risk factors [1]. Between 1990 and 2019, the global incidence of hypertensive disorders of pregnancy (HDP) grew from 16.30 million to 18.08 million, an increase of 10.92%. The number of fatalities from HDP reached 27.83 thousand in 2019, representing a 30.05% drop from 1990 to 2019. The age-standardized incidence rate (ASIR) fell from 579 per 100,000 people in 1990 to 463 in 2019, with an estimated annual percentage change (EAPC) of -0.68 and -2.38, respectively. The age-standardized death rate (ASDR) has similarly fallen [1].

The condition increases the prevalence of cardiovascular diseases (CVDs) in women postpartum, with cardiac dysfunction, hypertrophy, and ischemia. These changes persist in postpartum and have a strong relation to future cardiovascular morbidity and mortality, especially in severe cases and those with concomitant disorders [2].

This article aims to examine the rising incidence, risk factors, and global burden of hypertension during pregnancy, and to evaluate its long-term cardiovascular consequences for the affected women. By analyzing epidemiological trends and associated maternal health outcomes, the article seeks to highlight the significance of early identification, prevention, and management strategies to reduce maternal morbidity and mortality.

## Review

Classifications of hypertensive disorder in pregnancy

Chronic Hypertension

Chronic hypertension is characterized by increased systolic blood pressure (SBP) and/or diastolic blood pressure (DBP) before pregnancy or before 20 weeks of gestation [1].

Gestational Hypertension

If elevated SBP exceeds 140 mm Hg or DBP exceeds 90 mm Hg after 20 weeks of gestation, and if persistent postpartum blood pressure increases are present, it is considered chronic hypertension [1].

Preeclampsia Without Severe Features

It is known as recent hypertension after 20 weeks of gestation, accompanied by proteinuria in the absence of severe end-organ dysfunction or severe-range blood pressure (≥160/110 mmHg). The American College of Obstetrics and Gynecologists (ACOG) defines proteinuria as 300 mg or more/24-hour urine collection, a protein-to-creatinine ratio more than or equal to 0.3 mg/dL, or a dipstick reading of 2+ if quantitative techniques are unavailable. Preeclampsia can sometimes present without proteinuria and other diagnostic criteria, including thrombocytopenia, decreased hepatic functioning, severe right upper quadrant or abdominal discomfort, renal insufficiency, pulmonary edema, and a new-onset headache that does not respond to analgesics [1]. 

Preeclampsia With Serious Symptoms

It involves severe headaches, vision changes (flashing lights, blurriness), pain under the ribs (right side), persistent nausea/vomiting, sudden swelling (face, hands, feet), shortness of breath, confusion, seizures, and HELLP syndrome (high blood pressure, hemolysis, abnormal liver enzyme levels, and a low platelet count). HELLP commonly occurs in the third trimester and is associated with an increase in maternal morbidity and mortality. HELLP diagnosed with high lactate dehydrogenase (LDH), aspartate aminotransferase (AST), and alanine aminotransferase (ALT) values, as well as a platelet count of <100,000 × 10^9^/L. In 90% of cases, the major symptoms are right upper quadrant pain and general tiredness [1]. 

Chronic Hypertension Superimposed Preeclampsia

One to five percent of expectant mothers are affected, and 20-50% of them develop preeclampsia. The risk factors include Black race, obesity, smoking, having a history of preeclampsia, having a DBP of more than 100 mm Hg, and having persistent hypertension for more than four years. The frequency is substantially higher in women with secondary hypertension or organ failure (about 75%). Because it might be difficult to distinguish deteriorating chronic hypertension from superimposed preeclampsia, further vigilance is needed. In this group, superimposed preeclampsia usually manifests as a significant rise in liver enzyme levels or new-onset thrombocytopenia [1].

Pathophysiology and mechanistic links

Normal physiological responses to pregnancy include a higher metabolic rate, so that hemodynamic adjustments vary by each trimester and return to baseline after birth. Major maternal hemodynamic changes include higher cardiac output and plasma volume while decreasing arterial vascular resistance. Pregnancy is typically seen as a stressful condition, as inadequate adaptations result in mother and fetal illness and mortality. The first trimester of pregnancy, from fertilization to 13 weeks and six days, causes a 10% reduction in blood pressure. This is due to substantial peripheral vasodilation, which is caused in part by elevated levels of estrogen and progesterone. The concentration of relaxin rises and peaks near the end of the first trimester, generating a 50% rise in renal blood flow and glomerular filtration rate (GFR). Additional maternal hemodynamic adaptations occur to maintain adequate blood pressure, such as raised sympathetic and maternal baroreceptor sensitivity and triggering of the renin-angiotensin-aldosterone system, which counteract salt and water loss caused by renal vasodilation while increasing heart rate and cardiac output [1].

Arterial resistance gradually declines as relaxin levels fall to an intermediate level throughout the second trimester, which lasts from 14 to 27 weeks and six days of pregnancy. By 24 weeks, cardiac output has increased to 45% over baseline, and arterial pressures have reached a low point. Preeclampsia and gestational hypertension are linked to excessive sympathetic activity after 20 weeks, while the third trimester, 28 weeks to delivery, is marked by physiological anemia and increased blood pressure. The developing fetus benefits from improved placental perfusion made possible by this decrease in blood thickness. Plasma volume increases to 50% of nonpregnant levels near term, establishing a buffer against postpartum blood loss. The late trimester is when heart rate peaks, rising 20% to 25% over baseline. Systolic and diastolic blood pressure may increase by 10% to 15% and 15% to 25%, respectively, during vigorous labor. During the early stages of labor, cardiac output increases by 15%, and during the active phase, it increases by 25% [1].

Polymorphisms in genes associated with the renin-angiotensin system (RAS), endothelin (ET) system, inflammatory factors, and oxidative stress pathways enhance the risk of pregnancy-induced hypertension (PIH). RAS-related genes such as alanine-glyoxylate transaminase (*AGT*), angiotensin-converting enzyme (*ACE*), angiotensin II type 1/2 receptor (*AT1/2R*), and vitamin D Receptor (*VDR*) inhibit MMP-9, resulting in endothelial cell damage and placental failure. ET system-related genes such as *EDN1*, vascular endothelial growth factor (*VEGF*), and endothelial nitric oxide synthase (*eNOS*) increase nitric oxide (NO) as a vasodilator. Inflammation-related genes, such as *IL10* and *ERAP1/2*, reduce placental dysfunction by decreasing MMP-9 and angiotensin II. Paraoxonase 1 (*PON1*) and glutathione S-transferase mu 1 (*GSTM1*) mitigate oxidative stress by protecting low-density lipoproteins (LDLs) and lowering peroxide. Understanding the etiology of PIH can aid research into other hypertension and pregnancy-related illnesses [3].

Angiogenic imbalances, such as decreased VEGF and placental growth factor (PIGF) concentrations and increased soluble fms-like tyrosine kinase-1 (sFlt-1), are a hallmark of preeclampsia. As a result, there is less NO produced, which is necessary for vasodilation and vascular remodeling. Syncytio-trophoblast stress, which results in insufficient placentation, causes early-onset preeclampsia (EOPE) to develop before week 34 of pregnancy, whereas the placenta's expansion of its circulatory system causes late-onset preeclampsia (LOPE) to develop after week 34. Fetal development restriction brought on by protracted placental dysfunction is more frequently linked to EOPE [4]. Preeclampsia can be brought on by high blood pressure during pregnancy. Pregnant women with hypertension and chronic hypertension may experience preeclampsia, affecting up to 35% and 25%, respectively. Unknown, but it is linked to decreased placental perfusion, causing systemic vascular endothelial dysfunction. This is because the uterine spiral arteries' cytotrophoblast invasion is less successful. Endothelial dysfunction, sometimes referred to as preeclampsia syndrome, is caused by the resulting placental hypoxia, which sets off a series of inflammatory processes that upset the normal balance of angiogenic factors and result in aggregated platelets [5].

Evidence for long-term cardiovascular consequences

Hypertension and Ischemic Heart Disease

Several studies have revealed that women who have experienced hypertension during pregnancy are more vulnerable to acquiring CVD. However, the impact sizes varied greatly among different studies, and there is no thorough review of the existing information in the literature. Women having a history of HDP are more likely to suffer from CVD-related morbidity and mortality [6].

Heart Failure, Stroke, and Peripheral Artery Disease

It is a major worldwide cause of maternal mortality and morbidity, accounting for almost 10% of all pregnancies. These illnesses, including chronic hypertension, gestational hypertension, and preeclampsia or eclampsia, raise the risk of long-term stroke and account for 7.7% of maternal fatalities in the US. Predicting and avoiding these strokes remains difficult, with the greatest risk happening before or two days following birth [7].

Chronic Kidney Disease and Metabolic Syndrome

A higher incidence of maternal chronic kidney disease (CKD) postpartum is associated with preeclampsia. Preterm, recurrent, or preeclampsia made worse by prenatal obesity all raise this risk. The risk varies depending on the etiology of the CKD, with hypertensive, diabetic, and glomerular/proteinuric CKD having the highest risk. Gestational hypertension is also linked to an increased risk of CKD; however, the relationships are less strong than those for preeclampsia [8].

Timing and Magnitude of Risk: Early- Vs. Late-Onset Preeclampsia

Preeclampsia, a hypertensive disorder affecting up to 5% of pregnancies, can cause severe maternal complications like liver rupture, stroke, or even death. There is no consensus on the classification and diagnostic criteria, and differences between the American College of Obstetricians and Gynecologists (ACOG) and the International Society for the Study of Hypertension in Pregnancy (ISSHP) lead to adverse maternal and fetal outcomes [9].

Severity or Recurrence of HDP

Recurrence of hypertension illnesses during pregnancy is common, although it is not distinguished by the type of condition present in the first pregnancy. Overweight and weight gain during pregnancy have been linked to recurrent hypertensive difficulties in gestational hypertension-prone women. Early onset of hypertension is a risk variable independent of body weight [10].

Risk stratification and follow-up

According to the 2019 NICE guidelines, patients known with maternal disease or hypertension disorder are at risk of developing preeclampsia. On the other hand, nulliparous, age of ≥40, body mass index (BMI) of 35 kg/m^2^ or higher, family history of preeclampsia, carrying more than one fetus, or having skipped more than 10 years of pregnancy are all categorized as being at intermediate risk. These variables are reflected in the biggest analysis of risk factors, which was carried out by Bartsch et al. [11] and included approximately 25 million pregnancies from 92 studies. When aspirin prophylaxis is initiated before 16 weeks of pregnancy, preeclampsia is less likely to occur [12]. According to research, standard BP checks at in-person prenatal appointments were less successful than any alternative screening methods for hypertensive diseases during pregnancy. Morbidity and mortality from hypertensive diseases during pregnancy may be prevented; however, American Indian/Alaska Native people and Black people have uneven rates of adverse outcomes [13].

Recommended Postpartum Surveillance: BP Monitoring, Lipid Profiles, Glucose Testing

For HDP management, blood pressure monitoring at home (HBPM) may be a good substitute for in-office screening. In terms of reducing the risk of inducing labor, postpartum readmission, and boosting birth weight, this alternate approach has proven to be superior. HBPM performed as well as office monitoring in every other outcome assessed. Additionally, it was discovered that HBPM was safe for fetuses, neonates, and pregnant women [14]. A study found that although early postpartum lipid testing following HDP allows for the identification of women at elevated CV risk who may benefit from primary CVD preventive strategies, only approximately half of these high-risk women with HDP have their lipid profiles examined within the first four years after pregnancy [12]. Regardless of usual BMI or weight change from pre-pregnancy to postpartum, Chinese women with gestational diabetes mellitus (GDM) who had a history of HDP were at an increased risk for hypertension in the first five years after giving birth. Risk of hypertension in the first five years after birth was associated with a high pre-pregnancy BMI and weight gain of ≥7 kg from preconception to postpartum [15].

Preventive and therapeutic strategies

Research revealed that in the cost-effectiveness analysis of prenatal lifestyle treatments to date, lifestyle interventions are cost-efficient for lowering unfavorable maternal results during pregnancy, and measures for mothers are cost-saving. These findings are noteworthy because they expand on previous research from fewer participants that failed to show cost-effectiveness [16]. The way that people, families, and communities manage their physical, mental, social, and financial surroundings is referred to as their lifestyle. Self-concept and self-image are also influenced by lifestyle. Recent guidelines for managing hypertension recommend non-pharmacological treatments, and lifestyle modifications are considered the first-line management. Pregnant women with preeclampsia need specific lifestyle modifications in nutrition, physical exercise, smoking cessation, and stress reduction. Educational programs are also necessary for pregnant women with preeclampsia to ensure they follow the proper lifestyle for their health [17].

Preventing serious problems and mortality during pregnancy requires early detection and treatment of hypertension. Factors to consider include timing, whether hypertension is chronic or gestational, treatment timing, and recommended medications. ACOG recommends antihypertensive medication when blood pressure reaches a severe range (>160/110), but there is limited data on the benefits of mild-range hypertension treatment [18].

Antihypertensive Agents

During pregnancy, common antihypertensive medications used during pregnancy include vasodilators (e.g., hydralazine), calcium channel blockers - preferably dihydropyridines such as nifedipine - and central alpha-2 agonists (e.g., methyldopa). Labetalol, a combined beta-1 and alpha-1 blocker, is among the most commonly used agents. Diuretics are less commonly used during pregnancy due to their role in treating essential hypertension outside of pregnancy [19]. Peripheral-acting antiadrenergic agents like β-adrenergic, α-adrenergic, and calcium channel blockers may cause adverse effects like fatigue and exercise intolerance. Beta-blockers have been extensively studied without significant teratogenicity [19].

Central-acting antiadrenergic agents like methyldopa and clonidine are centrally acting antiadrenergic agents used to treat hypertension during pregnancy due to their safety and efficacy. Methyldopa depletes norepinephrine without affecting cardiac function or renal blood flow, converting it into a-methyl-norepinephrine, which stimulates adrenergic receptors, reducing sympathetic tone and blood pressure. Sleepiness, lightheadedness, exhaustion, sleeplessness, nightmares, and dry eyes are some of the adverse effects [19].

Vasodilators such as hydralazine are used for acute severe hypertension, typically in the hospital. Close monitoring is required because hydralazine can cause maternal hypotension and reflex tachycardia, and it may be associated with fetal tachycardia. It may result in myocardial ischemia or infarction in people who already have coronary artery disease [19]. Diuretics are hypertension medications that alter ion transport across the nephron cell membrane, increasing the kidneys' excretion of water and electrolytes during pregnancy. They aim to create volume depletion by interfering with sodium transport channels and dilatation of arterioles. The most widely used diuretic, thiazides, has not been consistently linked to either worsening intravascular depletion in preeclampsia patients or fetal growth limitation [19].

Angiotensin pathway inhibitors (ACE-I) are used to treat hypertension, heart failure, and diabetic nephropathy by suppressing the RAS system. They may, however, be fetotoxic, resulting in pulmonary and renal hypoplasia, oligoamnios, and fetal growth limitation secondary to hypotension and decreased placental circulation, leading to fetal mortality. The US Food and Drug Administration has mandated ACE-I warning statements during pregnancy since 1992 [19].

Prevention 

Pregnancy hypertension is a major problem, and aspirin has been advocated as the best pharmacological intervention for primary prevention of preeclampsia and other severe outcomes. Aspirin inhibits the cyclooxygenase enzyme in platelets and endothelial cells, disrupting the thromboxane-prostacyclin balance and favoring prostacyclin and its vasodilatory effects. It is prescribed to use low-dose aspirin 75-150 mg for individuals with substantial risk factors, estimating a 24% reduction in preeclampsia, 20% in growth restrictions, and 14% in preterm delivery. Antioxidants and 1.5 to 2 g/day calcium in areas with low dietary calcium intake are two nutritional supplements that have been examined for lowering the risk of preeclampsia [20].

Patients with low levels of education are vulnerable to having preterm births or dying as newborns. Women are tested for CVD at a lower rate than men, and the screening is frequently undertaken after diagnosis. This emphasizes the significance of knowing about high blood pressure in pregnant women. Patient education helps to reduce factors like CVD, check blood pressure, and cope with symptoms through a healthy lifestyle. To improve education, studies have used a wide range of educational interventions, such as smartphone apps, visual devices, and pictorial cards. Overall, the included studies revealed that educational intervention strategies had a good and significant impact on boosting pregnant women's understanding of HDP, which may help to prevent the disease's severe sequelae [21].

Fetal complications secondary to HDP

HDP is associated with a notable risk of preterm delivery. In many cases, the condition can compromise placental function or maternal health, making preterm birth more likely. Current estimates suggest that approximately 25-30% of pregnancies affected by HDP result in prematurity [22]. Studies have indicated that the most frequently observed fetal complications are intrauterine growth restriction (IUGR) and premature birth [23,24].

Another study reported that among 64 deliveries, 18.75% of newborns required NICU care for different reasons, while both intrauterine fetal death (IUFD) and neonatal mortality occurred in 1.56% of cases [25]. In comparison, another study found a 10% NICU admission rate, along with IUFD and neonatal death rates of 8.18% and 4.54%, respectively [24].

Early induction of labor versus expectant management in the context of HDP

Induction of labor for women with chronic or gestational hypertension has traditionally been debated because clinicians must balance the mother’s safety with the baby’s maturity. The WILL trial [26] included 403 pregnant women with either chronic or gestational hypertension who were nearing full term. They were randomly assigned to either have labor planned at 38+0 to 38+3 weeks (201 women) or to continue with usual care and deliver later unless a medical reason required earlier birth (202 women). On average, those in the planned-birth group delivered at 38.4 weeks, while those receiving usual care delivered at about 39.3 weeks, giving a difference of roughly six days between the two strategies, with a p-value reported as <0.001.

For maternal outcomes, the study looked at a combined measure of serious complications. This occurred in 27 women (13%) in the planned early-birth group and 24 women (12%) in the usual-care group, showing almost no difference between the two; the adjusted risk ratio is 1.16 with 95% CI 0.72-1.87 and p-value of 0.538. For babies, the key measure was whether they needed admission to a neonatal unit for at least four hours. This happened in 14 out of 201 infants (7%) in the planned-birth group and 14 out of 202 infants (7%) in the usual-care group, again indicating no disadvantage to earlier delivery. Cesarean delivery also did not increase with induction; instead, it occurred in 29% of the planned-birth group (58 of 201) compared with 36% in the usual-care group (72 of 202). These findings support scheduling birth around 38 weeks for women with well-managed hypertension; however, the trial’s smaller-than-intended sample size limits the strength of its conclusions.

The meta-analysis conducted by Jim Li et al. [27] demonstrates findings that are broadly consistent with those of the 2018 Cochrane review by Churchill and colleagues [28]. Both analyses report that immediate induction of labor is associated with a reduced risk of delivering small-for-gestational-age neonates, whereas delayed induction or expectant management appears to confer a lower risk of neonatal respiratory distress syndrome. Across the remaining outcomes examined in both reviews, no statistically meaningful differences were identified between the two approaches. Taken together, these findings indicate that the available evidence remains insufficient to conclusively support the superiority of either management strategy for hypertensive disorders in pregnancy.

Women who develop hypertensive disorders during pregnancy face a heightened risk of heart failure, whether ischemic or non-ischemic, extending from the short term into later life. Induction of labor in pregnant women with cardiac disease is guided by maternal functional status, the stability of the underlying cardiac condition, and fetal considerations [29-31]. Evidence from cardio-obstetric literature demonstrates that timing of delivery is individualized, based on maternal hemodynamic stability and disease severity, although several consistent indications for planned or early induction are described across open-access cohorts and reviews [29-32].

Induction is recommended when there is worsening heart failure, pulmonary edema, hypotension, or progressive symptoms despite optimal therapy. Both PPCM and structural heart disease reviews highlight that unstable hemodynamics warrant expedited delivery once maternal stabilization is achieved [30,31]. For women who remain asymptomatic and hemodynamically stable, induction of labor at ≥39 weeks is recommended to avoid unplanned intrapartum hemodynamic stress and improve neonatal outcomes. Evidence from congenital heart disease cohorts demonstrates higher neonatal morbidity with elective early-term delivery (37-38 weeks); therefore, planned induction at 39 weeks is preferred in stable patients [31,32].

Recurrence of HDP in the next pregnancies

Women who have experienced a hypertensive disorder in pregnancy face a higher chance of it recurring in a future pregnancy, although most will not be affected again. Overall, the likelihood of recurrence is about one in five, but the exact risk depends on the specific condition and how early or severely it occurred previously. Pre-eclampsia tends to have a higher recurrence risk, especially if it caused birth well before term (up to ~33% if delivery was at 28-34 weeks and ~23% at 34-37 weeks, compared with ~7% overall), while gestational hypertension generally has a lower chance of coming back (~9% overall and ~6-12% recurrence). Women with long gaps between pregnancies or with underlying chronic hypertension may also see increased risk. Beyond pregnancy-related concerns, a history of hypertensive disorders, particularly pre-eclampsia, is linked to a greater chance of developing long-term cardiovascular problems, making healthy lifestyle measures and appropriate medical follow-up important when planning future pregnancies [33].

These findings can help improve counseling for women planning another pregnancy by pinpointing those who are most likely to benefit from low-dose aspirin prophylaxis and closer monitoring in subsequent pregnancies [34].

Role of echocardiography in HDP 

Echocardiography is the main tool for assessing women with preexisting CVD during pregnancy. However, clear recommendations for routine screening in HDP are lacking. The American College of Obstetricians and Gynecologists advises prompt evaluation for women exhibiting warning signs, such as severely elevated blood pressure ≥160/110, with echocardiography considered one option for cardiac assessment [35]. Screening is additionally advised when there is suspicion of CVD, indicated by worrisome symptoms, moderately abnormal vital signs, unusual physical exam findings such as abnormal heart sounds, signs of heart failure, and abnormal pulse/rhythm, or the presence of multiple cardiac risk factors [36].

Blumenthal et al. [37] evaluated the effectiveness of this screening tool in a cohort of 846 women across two academic centers. Over half of the participants met at least moderate criteria for potential underlying CVD. Among those who completed the screening, 30% were found to have CVD. Detected abnormalities included left ventricular hypertrophy, systolic and diastolic dysfunction, and valvular lesions. Echocardiography was performed in 27.5% of the cohort. However, neither the International Society for the Study of Hypertension nor the American College of Obstetrics and Gynecology practice guidelines recommend routine echocardiographic screening for HDP [38,39]. 

Barriers and challenges

Maternal mortality remains a key barrier to meeting the Sustainable Development Goal of reducing the global maternal death ratio to less than 70 per 100,000 live births by 2030. However, there is a significant variation in maternal mortality rates, especially among low- and middle-income nations. Obstetric hemorrhage, maternal hypertension, sepsis, and abortion-related complications are among the leading causes of maternal mortality, with maternal hypertension considered the second leading cause of maternal death. Despite local and international measures and major improvements in maternal health coverage, progress toward eliminating unnecessary mortality in these mothers has been modest. Hypertensive disorders, particularly preeclampsia, are the most frequent medical condition during pregnancy and are associated with poor pregnancy outcomes. Preeclampsia causes around 15% of maternal mortality and serious maternal and perinatal pathologies in low- and middle-income countries [40].

Healthcare personnel, especially physicians and midwives, should improve awareness of hypertensive problems during pregnancy through educational messages and workshops. This can be accomplished through prenatal visits, community education campaigns, and media channels. Pregnancy care should be client-centered and adaptable, allowing for rapid diagnosis and improved health-care delivery. The research also underlines the importance of women with preeclampsia receiving support from partners, family, friends, and community members to reduce stigma and increase confidence in living positively with their disease [41].

## Conclusions

A serious health risk, hypertension in pregnancy can lead to endothelial damage and angiogenic imbalance with placental malfunction. These conditions raise the risk of metabolic syndrome, CKD, and CVS disease. To address this, multidisciplinary care models should be developed, involving obstetricians, cardiologists, nephrologists, primary care providers, and public health specialists. These models should extend beyond the perinatal period, provide structured follow-ups, and begin preventive cardiology in the postpartum period. Improving outcomes requires effective acute management, continuity of care, education, lifestyle interventions, and evidence-based pharmacological strategies.

## References

[1] Khedagi AM, Bello NA (2021). Hypertensive disorders of pregnancy. Cardiol Clin.

[2] Wang W, Xie X, Yuan T, Wang Y, Zhao F, Zhou Z, Zhang H (2021). Epidemiological trends of maternal hypertensive disorders of pregnancy at the global, regional, and national levels: A population-based study. BMC Pregnancy Childbirth.

[3] Wei W, Wang X, Zhou Y, Shang X, Yu H (2022). The genetic risk factors for pregnancy-induced hypertension: Evidence from genetic polymorphisms. FASEB J.

[4] Braunthal S, Brateanu A (2019). Hypertension in pregnancy: Pathophysiology and treatment. SAGE Open Med.

[5] Melchiorre K, Thilaganathan B, Giorgione V, Ridder A, Memmo A, Khalil A (2020). Hypertensive disorders of pregnancy and future cardiovascular health. Front Cardiovasc Med.

[6] Wu R, Wang T, Gu R (2020). Hypertensive disorders of pregnancy and risk of cardiovascular disease-related morbidity and mortality: A systematic review and meta-analysis. Cardiology.

[7] Wu P, Jordan KP, Chew-Graham CA (2020). Temporal trends in pregnancy-associated stroke and its outcomes among women with hypertensive disorders of pregnancy. J Am Heart Assoc.

[8] Barrett PM, McCarthy FP, Evans M (2020). Hypertensive disorders of pregnancy and the risk of chronic kidney disease: A Swedish registry-based cohort study. PLoS Med.

[9] Wójtowicz A, Zembala-Szczerba M, Babczyk D, Kołodziejczyk-Pietruszka M, Lewaczyńska O, Huras H (2019). Early- and late-onset preeclampsia: A comprehensive cohort study of laboratory and clinical findings according to the new ISHHP criteria. Int J Hypertens.

[10] Hjartardottir S, Leifsson BG, Geirsson RT, Steinthorsdottir V (2006). Recurrence of hypertensive disorder in second pregnancy. Am J Obstet Gynecol.

[11] Bartsch E, Medcalf KE, Park AL, Ray JG; High Risk of Pre-eclampsia Identification Group (2016). Clinical risk factors for pre-eclampsia determined in early pregnancy: Systematic review and meta-analysis of large cohort studies. BMJ.

[12] Fox R, Kitt J, Leeson P, Aye CY, Lewandowski AJ (2019). Preeclampsia: Risk factors, diagnosis, management, and the cardiovascular impact on the offspring. J Clin Med.

[13] Henderson JT, Webber EM, Thomas RG, Vesco KK (2023). Screening for hypertensive disorders of pregnancy: Updated evidence report and systematic review for the US Preventive Services Task Force. JAMA.

[14] Albadrani M, Tobaiqi M, Al-Dubai S (2023). An evaluation of the efficacy and the safety of home blood pressure monitoring in the control of hypertensive disorders of pregnancy in both pre and postpartum periods: A systematic review and meta-analysis. BMC Pregnancy Childbirth.

[15] Wen C, Metcalfe A, Anderson TJ, Johnson JA, Sigal RJ, Nerenberg KA (2019). Measurement of lipid profiles in the early postpartum period after hypertensive disorders of pregnancy. J Clin Lipidol.

[16] Wang L, Leng J, Liu H (2017). Association between hypertensive disorders of pregnancy and the risk of postpartum hypertension: A cohort study in women with gestational diabetes. J Hum Hypertens.

[17] Bailey C, Skouteris H, Harrison CL (2020). Cost effectiveness of antenatal lifestyle interventions for preventing gestational diabetes and hypertensive disease in pregnancy. Pharmacoecon Open.

[18] Ali S, Abdrabbo R, Shalaby N, Abdel Ati I (2022). Effect of lifestyle modification guidelines on maternal and fetal outcomes among pregnant women with mild preeclampsia. Port Said Sci J Nurs.

[19] Leavitt K, Običan S, Yankowitz J (2019). Treatment and prevention of hypertensive disorders during pregnancy. Clin Perinatol.

[20] Sinkey RG, Battarbee AN, Bello NA, Ives CW, Oparil S, Tita AT (2020). Prevention, diagnosis, and management of hypertensive disorders of pregnancy: A comparison of international guidelines. Curr Hypertens Rep.

[21] Gholami K, Norouzkhani N, Kargar M (2022). Impact of educational interventions on knowledge about hypertensive disorders of pregnancy among pregnant women: A systematic review. Front Cardiovasc Med.

[22] Jain L (1997). Effect of pregnancy-induced and chronic hypertension on pregnancy outcome. J Perinatol.

[23] Gavali S, Patil A, Gavali U (2021). Study of fetomaternal outcome in patients with pregnancy induced hypertension at Sangli district. MedPulse Int J Gynaecol.

[24] Lama S, Gurung P, Malla AP (2023). Pregnancy induced hypertensive disorders among patients admitted to the Department of Obstetric and Gynecology in a tertiary care centre: A descriptive cross-sectional study. JNMA J Nepal Med Assoc.

[25] Patel R, Baria H, Patel HR, Nayak S (2017). A study on pregnancy induced hypertension and foetal outcome among patients with PIH at tertiary care hospital, Valsad. Int J Community Med Public Health.

[26] Magee LA, Kirkham K, Tohill S (2025). Correction: Determining optimal timing of birth for women with chronic or gestational hypertension at term: The WILL (When to Induce Labour to Limit risk in pregnancy hypertension) randomised trial. PLoS Med.

[27] Li J, Shao X, Song S, Liang Q, Liu Y, Qi X (2020). Immediate versus delayed induction of labour in hypertensive disorders of pregnancy: A systematic review and meta-analysis. BMC Pregnancy Childbirth.

[28] Churchill D, Duley L, Thornton JG, Moussa M, Ali HS, Walker KF (2018). Interventionist versus expectant care for severe pre-eclampsia between 24 and 34 weeks' gestation. Cochrane Database Syst Rev.

[29] Sanghavi M, Rutherford JD (2014). Cardiovascular physiology of pregnancy. Circulation.

[30] Gongora MC, Wenger NK (2015). Cardiovascular complications of pregnancy. Int J Mol Sci.

[31] Roos-Hesselink J, Baris L, Johnson M (2019). Pregnancy outcomes in women with cardiovascular disease: Evolving trends over 10 years in the ESC Registry Of Pregnancy And Cardiac disease (ROPAC). Eur Heart J.

[32] Mok T, Woods A, Small A (2022). Delivery timing and associated outcomes in pregnancies with maternal congenital heart disease at term. J Am Heart Assoc.

[33] (2023). National Institute for Health and Care Excellence. Hypertension in Pregnancy: Risk of Recurrence of Hypertensive Disorders of Pregnancy (Visual Summary). https://www.nice.org.uk/guidance/ng133/resources/risk-of-recurrence-of-hypertensive-disorders-of-pregnancy-pdf-313945610007.

[34] Adu-Bonsaffoh K, Tamma E, Nwameme AU, Browne JL (2022). Health professionals' perspectives on clinical challenges in managing hypertensive disorders of pregnancy and recommendations for improving care: A multi-center qualitative study. Front Glob Womens Health.

[35] Briller JE (2022). Echocardiographic screening in hypertensive pregnancy disorders: Probing the window of opportunity. J Am Coll Cardiol.

[36] American College of Obstetricians and Gynecologists' Presidential Task Force on Pregnancy and Heart Disease and Committee on Practice Bulletins—Obstetrics (2019). ACOG practice bulletin no. 212: Pregnancy and heart disease. Obstet Gynecol.

[37] Blumenthal EA, Crosland BA, Senderoff D (2020). California cardiovascular screening tool: Findings from initial implementation. AJP Rep.

[38] (2020). Gestational hypertension and preeclampsia: ACOG practice bulletin, Number 222. Obstet Gynecol.

[39] Brown MA, Magee LA, Kenny LC (2018). Hypertensive disorders of pregnancy: ISSHP classification, diagnosis, and management recommendations for international practice. Hypertension.

[40] Lailler G, Grave C, Gabet A (2023). Recurrence of hypertensive disorders of pregnancy: Results from a nationwide prospective cohort study (CONCEPTION). BJOG.

[41] Muhindo G, Muwanguzi P, Moore S, Kaddumukasa M, Sajatovic M, Mbalinda SN (2025). Barriers and enablers of early health-seeking behaviour among women with preeclampsia and eclampsia: A qualitative study at a National referral hospital in Uganda. BMC Pregnancy Childbirth.

